# Comparative transcriptomics uncovers alternative splicing and molecular marker development in radish (*Raphanus sativus* L.)

**DOI:** 10.1186/s12864-017-3874-4

**Published:** 2017-07-03

**Authors:** Xiaobo Luo, Liang Xu, Dongyi Liang, Yan Wang, Wei Zhang, Xianwen Zhu, Yuelin Zhu, Haiyan Jiang, Mingjia Tang, Liwang Liu

**Affiliations:** 1 National Key Laboratory of Crop Genetics and Germplasm Enhancement, College of Horticulture, Nanjing Agricultural University, Nanjing, 210095 People’s Republic of China; 2 Department of Plant Sciences, North Dakota State University, Fargo, ND 58108 USA

**Keywords:** Radish, Transcriptome, Alternative splicing (AS), Single nucleotide polymorphism (SNP), Insertion/deletion (InDel), Genetic diversity

## Abstract

**Background:**

Alternative splicing (AS) plays important roles in gene expression and proteome diversity. Single nucleotide polymorphism (SNP) and insertion/deletion (InDel) are abundant polymorphisms and co-dominant inheritance markers, which have been widely used in germplasm identification, genetic mapping and marker-assisted selection in plants. So far, however, little information is available on utilization of AS events and development of SNP and InDel markers from transcriptome in radish.

**Results:**

In this study, three radish transcriptome datasets were collected and aligned to the reference radish genome. A total of 56,530 AS events were identified from three radish genotypes with intron retention (IR) being the most frequent AS type, which accounted for 59.4% of the total expressed genes in radish. In all, 22,412 SNPs and 9436 InDels were identified with an average frequency of 1 SNP/17.9 kb and 1 InDel/42.5 kb, respectively. A total of 43,680 potential SSRs were identified in 31,604 assembled unigenes with a density of 1 SSR/2.5 kb. The ratio of SNPs with nonsynonymous/synonymous mutations was 1.05:1. Moreover, 35 SNPs and 200 InDels were randomly selected and validated by Sanger sequencing, 83.9% of the SNPs and 70% of the InDels exhibited polymorphism among these three genotypes. In addition, the 15 SNPs and 125 InDels were found to be unevenly distributed on 9 linkage groups. Furthermore, 40 informative InDel markers were successfully used for the genetic diversity analysis on 32 radish accessions.

**Conclusions:**

These results would not only provide new insights into transcriptome complexity and AS regulation, but also furnish large amount of molecular marker resources for germplasm identification, genetic mapping and further genetic improvement of radish in breeding programs.

**Electronic supplementary material:**

The online version of this article (doi:10.1186/s12864-017-3874-4) contains supplementary material, which is available to authorized users.

## Background

The advent of next-generation sequencing (NGS) has greatly advanced our opportunities to obtain abundant sequences data by resequencing plant genome and transcriptome in greater depth [1, 2]. RNA-Seq technology had been used to rapidly isolate and identify alternative splicing (AS) [3, 4], novel transcripts [5] and structural variations [6]. Surprisingly, the transcriptome data offers rich resources to identify and develop large number of single nucleotide polymorphism (SNP) and insertion/deletion (InDel) markers based on comparing transcriptome sequences to the reference genome sequences [7, 8]. This approach offers a direct, reliable and high-efficient strategy for identification and development of SNPs and InDels using data mining by bioinformatic methods. Recently, using comparative transcriptomic analysis, a large number of SNP and InDel markers have been identified in many plant species including *Arabidopsis* [7], *Brassica napus* [9], rice [10] and tomato [11]. However, a limited number of SNP and InDel markers have been developed in transcriptome data of radish [12].

Molecular markers related to phenotypes provide us with valuable tools for elucidation of genetic variations. In the past two decades, several molecular marker systems, such as restriction fragment length polymorphism (RFLP), random amplified polymorphic DNA (RAPD), amplified fragment length polymorphism (AFLP) and simple sequence repeats (SSR) markers had been widely employed in genetic diversity analysis, and genetic mapping studies in many plant species [10, 13–15]. Recently, SNP and InDel markers has increasingly being received attention owing to their high frequency, wide distribution, co-dominant inheritance and abundant DNA polymorphisms [16, 17]. Previous studies indicated that the SNPs and InDels within or near the coding sequences could be influenced by certain phenotype, transcription factor binding and alternative splicing [18, 19]. SNP and InDel markers have been extensively utilized in several crop improvement programs including genetic diversity analysis, quantitative trait locus (QTL) mapping and genome-wide association analyses (GWAS) [20, 21].

Alternative splicing, generating multiple transcript variants from a single pre-mRNA and increasing the transcriptome complexity, is one of the most important regulatory mechanisms for gene expression and functional diversity of proteome [22, 23]. In plants, it has been estimated that 60% of *Arabidopsis* genes [24], 33% of rice genes [23], 40% of soybean genes [25] and 59.3% of tomato genes [26] could be alternatively spliced. Exon-skipping (ES) events were the predominant AS type in animals, while intron retention (IR) was more frequent in plants [27]. Moreover, AS played crucial roles in regulation of biological functions, particularly in developmental processes and stress responses [24]. Genome-wide analysis of AS events had been surveyed in tomato, which showed that the splice variants of multi-exon genes in the seedlings and flowers were lower than those in early growth fruits [26]. In six teosinte and 10 maize transcriptomes, a large number of genes were induced in AS complexity during domestication from teosinte to maize [3]. These results provided useful information for identification and characterization of AS in other plant species.

Radish (*Raphanus sativus* L., 2n = 2× = 18) is an economically annual or biennial root vegetable crop of the Brassicaceae family. Although a set of SNP and/or InDel markers had been developed from the public radish EST database or whole genome resequencing data [28, 29], the number of SNP and InDel markers are far from saturated for linkage mapping and association study in radish. Recently, the release of radish reference genome [30] and availability of de novo transcriptome datasets [31–33] generated a great number of transcriptome sequences, which provided useful sequence information for identification and development of SNP, InDel and SSR markers in radish.

To better systematically characterize the overall transcriptional landscape in radish, three available radish transcriptomes data were collected in this study. The alignment of the short reads to the radish genome dataset were conducted for identification of AS events, novel mRNA transcripts as well as SNP, InDel and SSR markers among these three radish transcriptomes. Additionally, the frequency, distribution and polymorphic of SNPs and InDels were estimated and characterized in three radish genotypes. The newly developed InDel markers were applied to investigate the genetic diversity among 32 accessions. The outcomes of this study could enhance our understanding of radish gene structure and facilitate marker-assisted selection and genetic improvement of some important horticulture traits in radish breeding programs.

## Results

### Overview of the RNA-Seq data

**Fig. 1:**
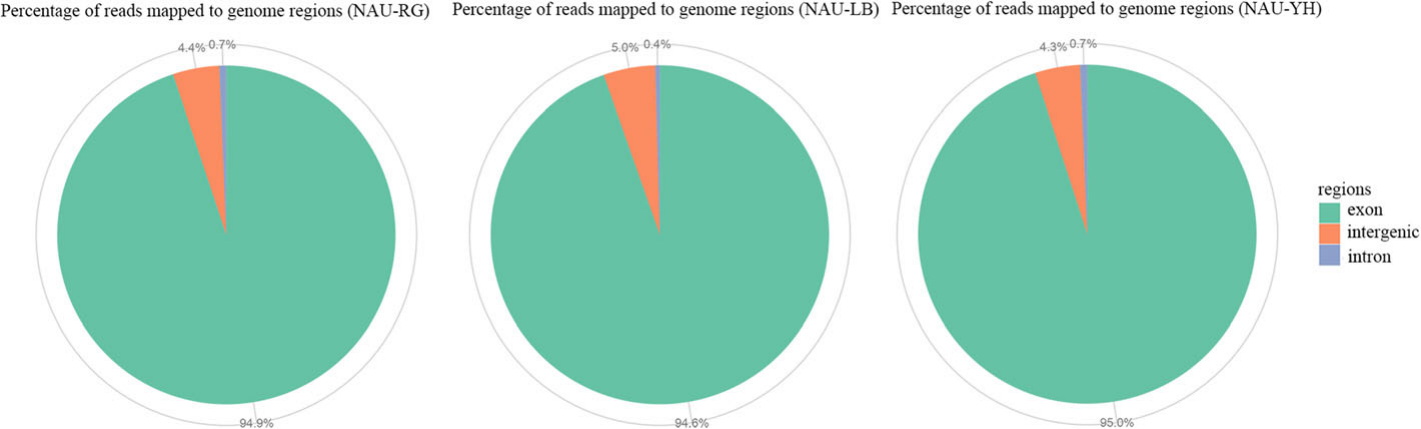
Overview of RNA-Seq reads mapping to radish reference genome. The percentage of clean reads mapped to reference genome regions in the NAU-RG, NAU-LB and NAU-YH libraries

To identify alternative splicing events and molecular marker in radish, three transcriptome datasets of the radish genotypes ‘NAU-RG’, ‘NAU-LB’ and ‘NAU-YH’ were collected and used for further analysis. After filtering the low-quality reads and adapter sequences, 64,000,056, 49,631,196 and 50,386,238 clean reads were obtained in ‘NAU-RG’, ‘NAU-LB’ and ‘NAU-YH’ libraries, respectively, from which 50,047,249 (78.2%), 34,770,143 (70.06%) and 37,512,238 (74.45%) clean reads in ‘NAU-RG’, ‘NAU-LB’ and ‘NAU-YH’ libraries, respectively, were mapped onto the reference genome sequence of radish (Table 1). Totally, 49,481,475 (77.31%), 34,413,111 (69.34%) and 37,111,575 (73.65%) clean reads in the ‘NAU-RG’, ‘NAU-LB’ and ‘NAU-YH’ libraries, respectively, were mapped to unique genome locations. The uniquely mapped reads were aligned to the exon, intron and intergenic regions of reference genome (Fig. 1).

**Table 1:** Summary of clean reads and mapped to the radish reference genome

Sample name	NAU-RG	NAU-LB	NAU-YH
Total reads	64,000,056	49,631,196	50,386,238
Total mapped	50,047,249 (78.2%)	34,770,143 (70.06%)	37,512,238 (74.45%)
Multiple mapped	565,774 (0.88%)	357,032 (0.72%)	400,663 (0.8%)
Uniquely mapped	49,481,475 (77.31%)	34,413,111 (69.34%)	37,111,575 (73.65%)
Read-1	24,828,322 (38.79%)	17,299,967 (34.86%)	18,743,053 (37.2%)
Read-2	24,653,153 (38.52%)	17,113,144 (34.48%)	18,368,522 (36.46%)
Reads map to ‘+’	24,760,998 (38.69%)	17,207,989 (34.67%)	18,602,410 (36.92%)
Reads map to ‘-’	24,720,477 (38.63%)	17,205,122 (34.67%)	18,509,165 (36.73%)
Non-splice reads	37,822,982 (59.1%)	25,258,482 (50.89%)	27,697,799 (54.97%)
Splice reads	11,658,493 (18.22%)	9,154,629 (18.45%)	9,413,776 (18.68%)

Multiple mapped, number of reads mapped to multiple sites; uniquely mapped, number of reads mapped to one site only; Read-1 and Read-2, reads from the two separate lanes; Reads mapped to ‘+’ or ‘−’, mapped to ‘+’ or ‘−’ strands of DNA; Splice reads, number of reads mapped to splice sites

### Identification of AS events and discovery of novel mRNA transcripts

**Fig. 2:**
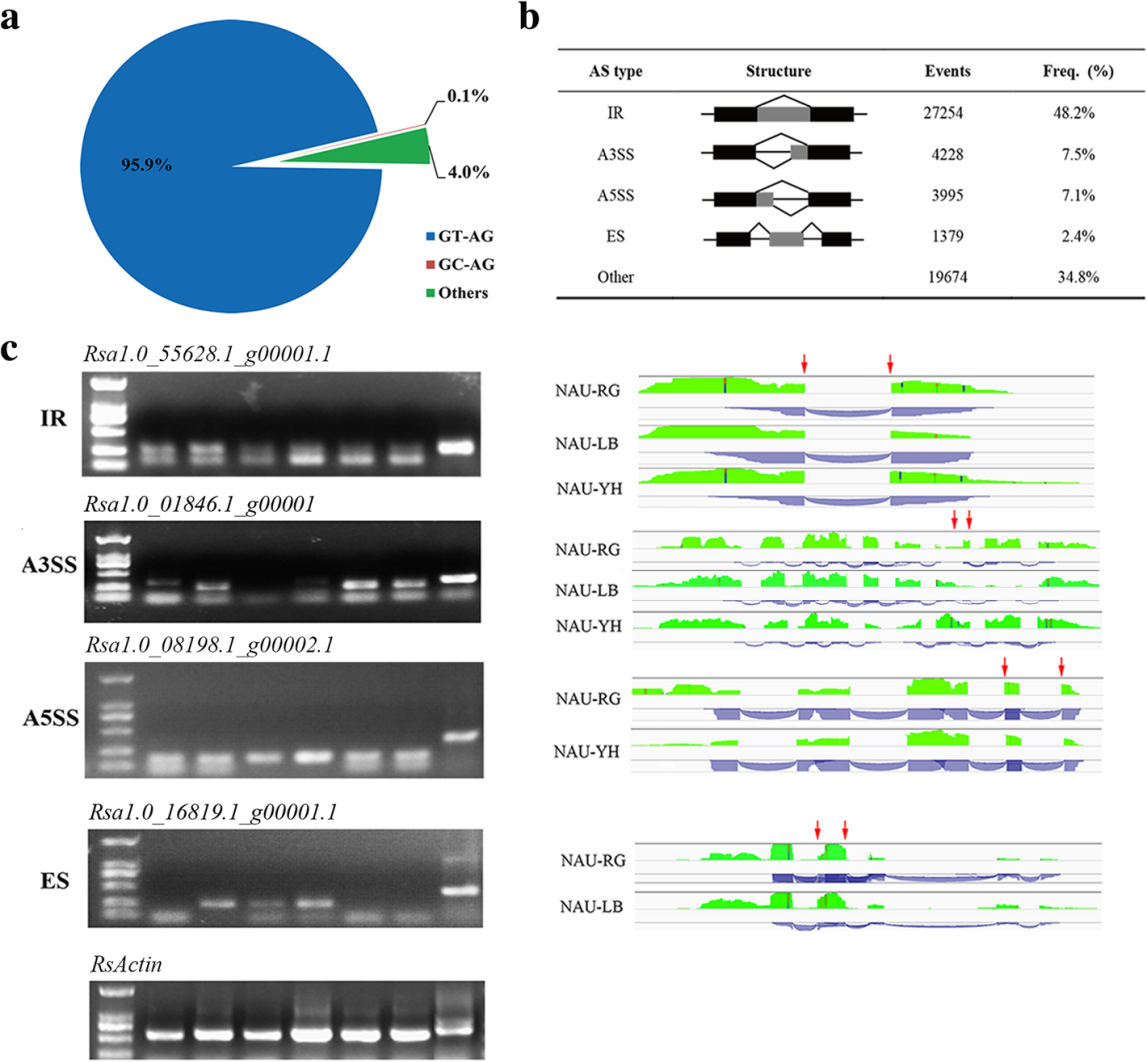
Overall characteristics of AS events**. a** The proportions for the three types of dinucleotides at the splicing border. **b** Number and frequency of the different AS types. (IR) Intron retention, (A3SS) alternative 3′ splice site, (A5SS) alternative 5′ splice site, (ES) exon skipping. **c** Four genes representing four types of AS events were validated by RT-PCR. The lanes from left to right are DNA ladder, leaf and root of ‘NAU-RG’, ‘NAU-LB’, ‘NAU-YH’, and genomic DNA of ‘NAU-YH’. The RNA-Seq read density of the four genes was viewed by IGV browser (green peaks). The red arrows indicate alternative splice sites. The blue arcs indicate splice junction reads that support the junctions

In all, 170,086 unique splice junctions sites were identified using TopHat program [34] (Additional file 1: Table S2); among which 131,106 (77.1%) were annotated in the radish genome and 38,980 (22.9%) were novel splice junctions. As shown in Additional file 1: Table S2, the ‘NAU-LB’ had the largest number of junctions, followed by ‘NAU-YH’ and ‘NAU-RG’. Among the dinucleotides at the intron border, a total of 218,574 GT-AG splice sites (95.9%), 163 GC-AG splice sites (0.1%), and 9256 splice sites of other types (4.0%) were identified (Fig. 2a).

To analyze the AS events in radish, the ASTALAVISTA software was employed to extract the AS events and classify different types of alternative splicing [35]. In total, 56,530 AS events were identified in three transcriptome datasets. These AS events were distributed in 36,546 genes, which accounted for 59.4% of the total radish genes, with an average of 1.68 AS events per gene. In this study, 25.0% of the radish AS-related genes undergo multiple AS events resulting in a variety of transcripts from a single gene (Additional file 1: Figure S1). The splice junctions of these genes were used to identify and categorize the AS events into five main types of AS events as intron retention (IR), exon skipping (ES), alternative 3′ splice site donor (A3SS), alternative 5′ splice site acceptor (A5SS) and other events. Among the different AS types, IR events were the predominant AS type (27,254, 48.2%), followed by other (19,674, 34.8%), A3SS (4228, 7.5%), and A5SS (3995, 7.1%) events (Fig. 2b, Additional file 2). To verify the accuracy of AS events, a reverse transcription-PCR (RT-PCR) analysis was performed on the leaf and root of ‘NAU-RG’, ‘NAU-LB’ and ‘NAU-YH’, respectively. Nine AS events from nine genes were exhibited to produce splice variants, which were consistent with the RNA-Seq data results. The RT-PCR results indicated that the expression of AS events was tissue-specific, for example, *Rsa1.0_01846.1_g00001.1* was expressed in the root of ‘NAU-LB’, while not expressed in the leaf (Fig. 2c, Additional file 1: Table S3).

**Fig. 3:**
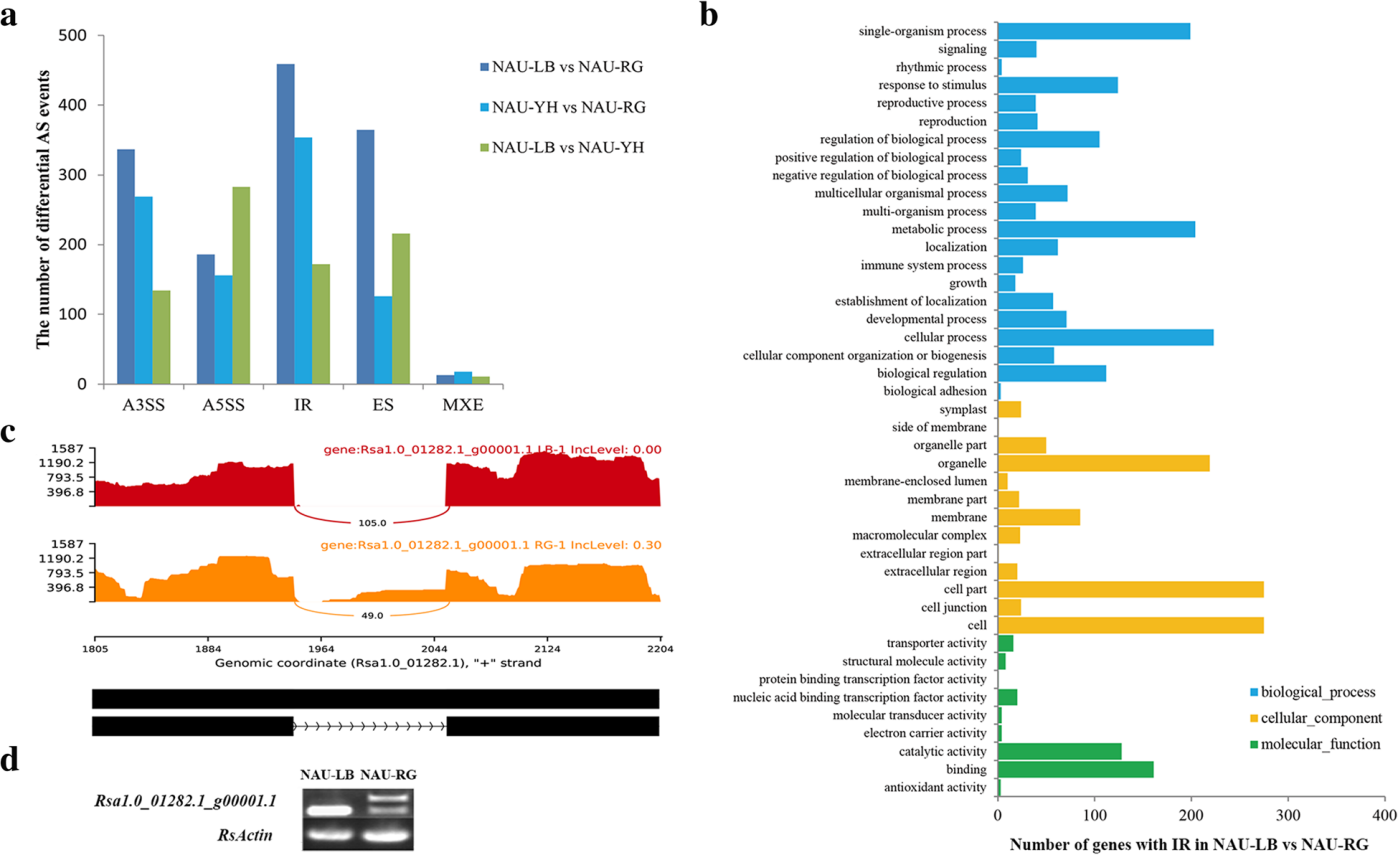
Characterization of differential splicing events in three genotypes. **a** Distribution of differential splicing events in three pairwise comparisons. **b** Gene ontology classification of genes with differential IR events in ‘NAU-LB’ vs ‘NAU-RG’. **c** Reads distribution of *Rsa1.0_01282.*1_g00001.1 derived from RNA-Seq data of ‘NAU-LB’ and ‘NAU-RG’. The horizontal and vertical axis represents per-base expression and genomic coordinates, respectively. mRNA isoform quantified is given at the bottom, black box and line with arrow heads represents exon and intron, respectively. **d** Validation of *Rsa1.0_01282.1_g00001.1* with differential IR event in the ‘NAU-LB’ and ‘NAU-RG’ comparison by RT-PCR

To detect the differential splicing events, the AS events consisted of A3SS, A5SS, IR, ES and mutually exclusive exons (MXE) were analyzed using the Multivariate Analysis of Transcript Splicing (MATS) program [36]. The maximum number of differential AS (1360) was identified in ‘NAU-LB’ vs ‘NAU-RG’, of which RI (459 events in 422 genes) possessed the predominant events (Fig. 3a, Additional file 3). Gene ontology (GO) enrichment analysis showed that a total of 336 (79.6%) differentially spliced genes were assigned into 43 GO terms in the comparison between ‘NAU-LB’ and ‘NAU-RG’ (Fig. 3b). In terms of three main categories, ‘cellular process’, ‘cell’ and ‘binding’ represented the most abundant GO terms in biological process, cellular component and molecular function, respectively. In ‘NAU-LB’ and ‘NAU-RG’ comparison, the *Rsa1.0_01282.1_g00001.1* with IR event was selected and validated (Fig. 3c). A novel isoform was found in ‘NAU- RG’, but not in ‘NAU- LB’ by RT-PCR (Fig. 3d). In this study, the novel and unknown genes were defined as novel transcripts, a total of 10,657 novel transcripts were detected based on the transcriptome data (Additional file 4). The newly identified transcripts would be useful for updating the radish genome annotation.

### Identification and characterization of SNPs, InDels and SSRs in radish

**Fig. 4:**
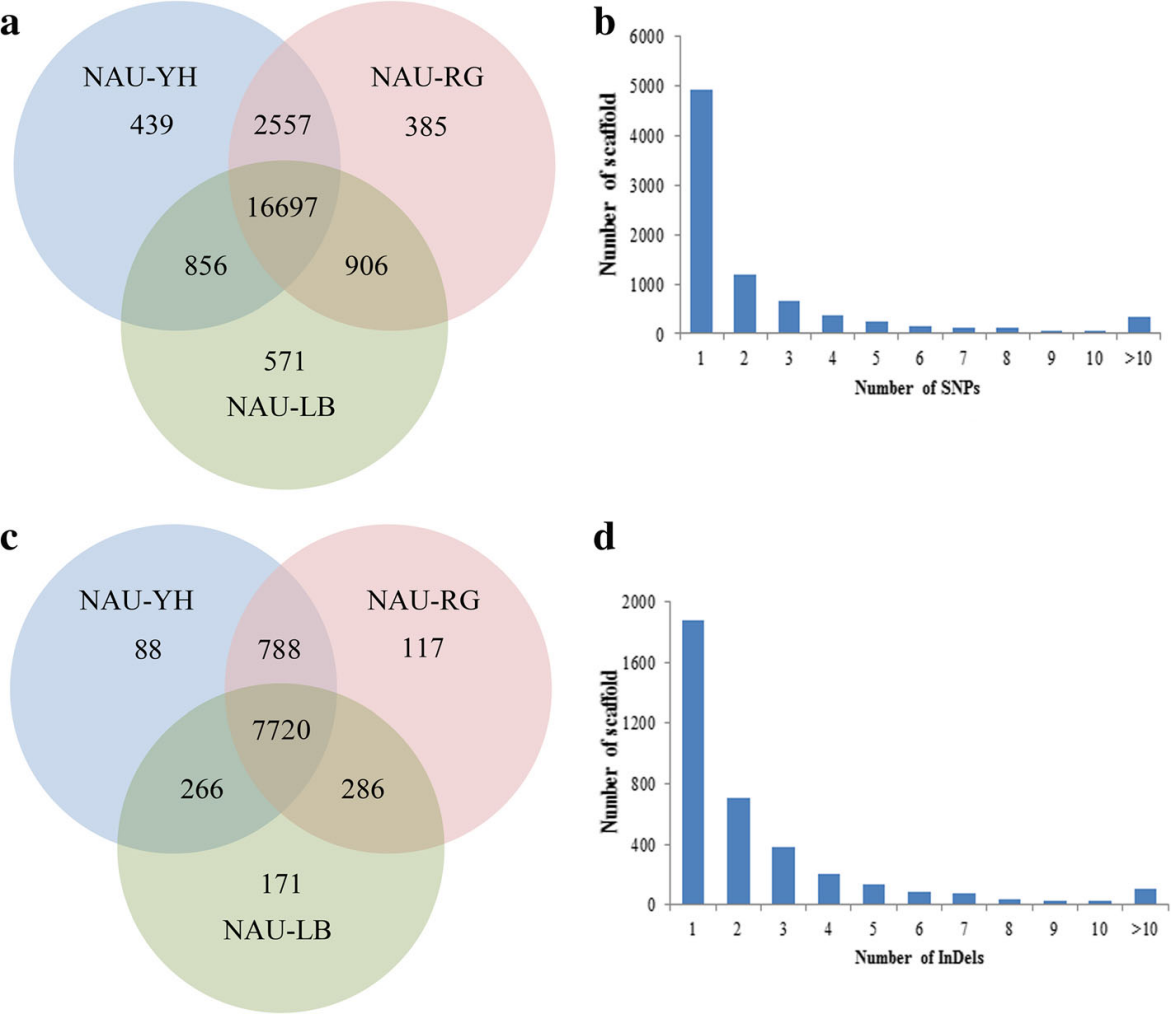
Summary of SNPs and InDels between three genotypes. Number of SNPs (**a**) and InDels (**c**) presented in three genotypes; Number of SNPs (**b**) and InDels (**d**) on the scaffold. The intersecting portions represent the common loci between three genotypes

To systematically identify genetic variations, three radish transcriptomes were utilized for SNPs and InDels discovery using Genome Analysis Toolkit [37]. Overall, 22,412 putative SNPs were detected among these three genotypes with an average frequency of 1 SNP/17.9 kb in comparison with the reference sequence (Additional file 5). Of these, 19,013, 20,547 and 20,551 SNPs were identified in ‘NAU-LB’, ‘NAU-RG’ and ‘NAU-YH’, respectively. A total of 16,997 SNPs were shared by all three genotypes, while 439, 571 and 385 SNPs were specific for ‘NAU-YH’, ‘NAU-LB’ and ‘NAU-RG’, respectively (Fig. 4a). The identified SNPs were distributed on the 8248 scaffolds, and the majority of scaffolds only had one SNP (Fig. 4b).

SNPs were further classified based on their zygosity and nucleotide substitution. Between these three pairwise comparisons, 14,583 SNPs (13,241 homozygous and 1342 biallelic SNPs) detected between ‘NAU-RG’ and ‘NAU-YH’ were more than those in the other two transcriptome comparisons (Table 2). A total of 3838 (17.1%) and 3209 (14.3%) homozygous SNPs were presented in ‘NAU-LB’ vs ‘NAU-RG’ comparison and ‘NAU-LB’ vs ‘NAU-YH’ comparison, respectively. There were 1342 homozygous SNPs between ‘NAU-RG’ vs ‘NAU-YH’ comparison, which comprised only 6.0% of the total SNPs, indicating that the self-incompatible ‘NAU-YH’ presented a larger degree of heterozygosity than other two accessions (Table 2). Moreover, the frequency of transitions (58.5%) was higher than transversions (41.5%) with transition to transversion ratio (Ts/Tv) of 1.41:1 (Table 2). Across all pairwise comparisons, the Ts/Tv of bi-allelic SNPs was slightly more than the homozygous SNPs, the overall Ts/Tv being relatively stable between genotypes with a range from 1.29 to 1.4.

**Table 2:** SNPs zygosity and corresponding transition to transversion ratio (Ts/Tv) in pairwise transcriptome comparisons

	Number (%)	C/T (%)	A/G (%)	C/G (%)	A/T (%)	C/A (%)	T/G (%)	Ts/Tv
		Transition	Transition	Transversion	Transversion	Transversion	Transversion	
All SNPs	22,412(100)	6643(29.6)	6474(28.9)	1992(8.9)	2661(11.9)	2383(10.6)	2259(10.1)	1.41
Homozygous								
NAU-LB and NAU-RG	3838 (17.1)	1085 (4.8)	1188 (5.3)	403 (1.8)	423 (1.9)	418 (1.9)	319 (1.4)	1.35
NAU-LB and NAU-YH	3209 (14.3)	940 (4.2)	887 (4.0)	316 (1.4)	392(1.7)	346 (1.5)	328 (1.5)	1.32
NAU-RG and NAU-YH	1342 (6.0)	378 (1.7)	377 (1.7)	136 (0.6)	167 (0.7)	155 (0.7)	129 (6.0)	1.29
Biallelic								
NAU-LB and NAU-RG	9930 (44.3)	2940 (13.1)	2809(12.5)	1226(5.5)	908 (4.1)	1032 (4.6)	1015 (4.5)	1.38
NAU-LB and NAU-YH	9559 (42.7)	2808 (12.5)	2748 (12.3)	849 (3.8)	1179 (5.3)	1004 (4.5)	971 (4.3)	1.33
NAU-RG and NAU-YH	13,241 (59.1)	3913 (17.5)	3802 (17.1)	1616 (7.2)	1163 (5.2)	1335 (6.0)	1412 (6.3)	1.4

Compared to the reference sequence, a total of 9436 InDels were identified with an average density of 1 InDel/42.5 kb (Fig. 4c, Additional file 6). These InDels were distributed on 3661 scaffolds with the length ranged from 1 to 13 bp, and for 1873 InDels (51.2%), each was located in one scaffold (Fig. 4d). The single-nucleotide InDels (82.7%) was the dominant type, followed by bi- (14.7%) and tri- (1.3%) nucleotide InDels (Additional file 1: Table S4). A comparative analysis showed that the maximum (8508) and minimum (7986) number of InDels were presented in ‘NAU-RG’ vs ‘NAU-YH’ and ‘NAU-LB’ vs ‘NAU-YH’ comparison, respectively, of which 2066 (21.9%) and 2835 (30.0%) InDels were homozygous in ‘NAU-RG’ vs ‘NAU-YH’ and ‘NAU-LB’ vs ‘NAU-YH’ comparison, respectively.

**Fig. 5:**
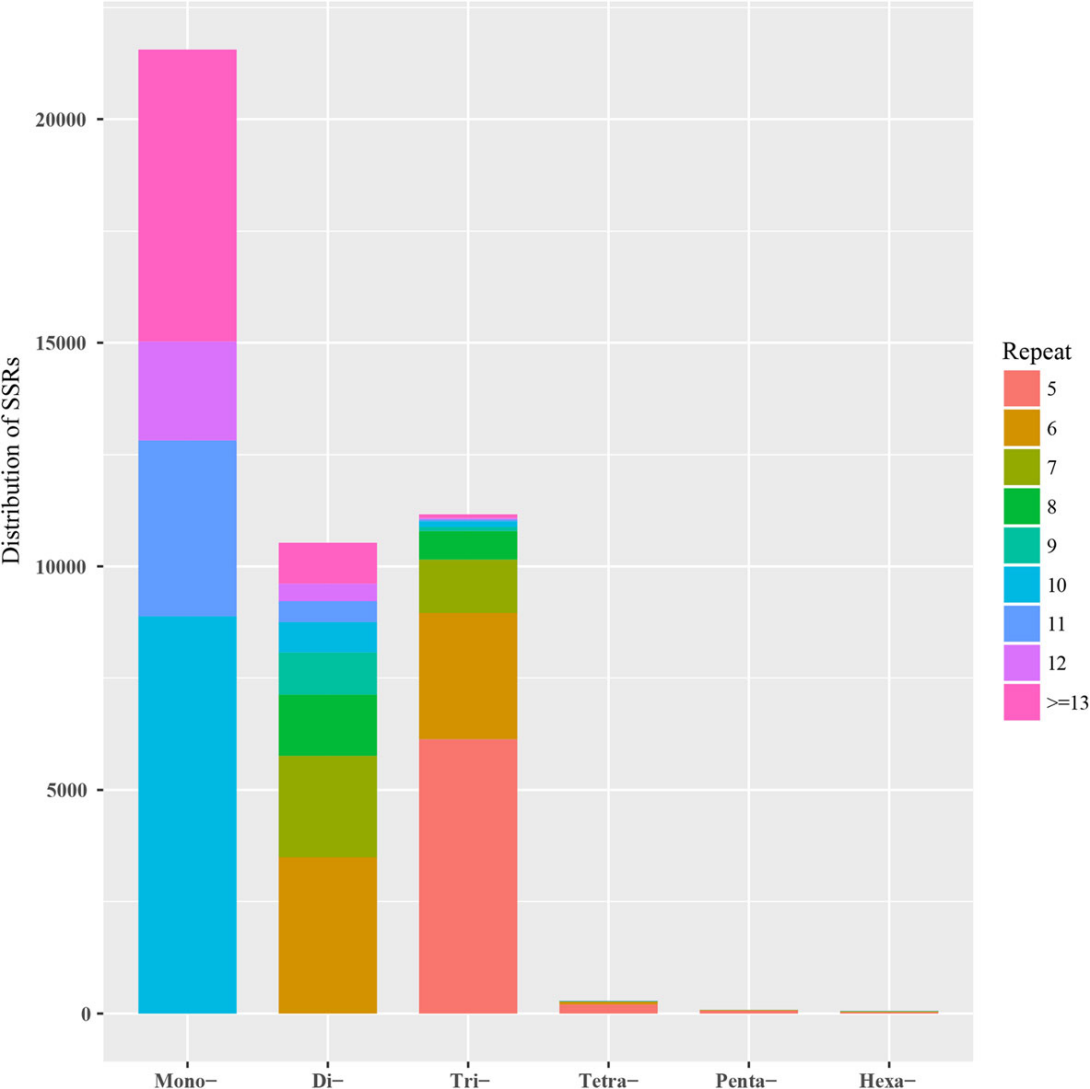
Distribution of microsatellites with different motif lengths in radish

To identify the potential SSRs, a total of 104,801 assembled unigenes were obtained from three transcriptome datasets. In total, 43,680 potential SSRs were identified in 31,604 unigenes with a density of 1 SSR/2.5 kb, among which 8009 (25.0%) possessed more than one SSR (Additional file 7). Among the identified SSRs, the mono-nucleotide repeats were the most abundant (21,599, 49.4%), followed by tri- (10,529, 25.6%), di- (11,164, 24.1%), and tetra- (287, 0.7%) nucleotide repeats, respectively (Fig. 5). For the mono-nucleotide motifs, A/T was the primary motif. AG/CT and GA/TC were the most common motifs among the di-nucleotide motifs (Additional file 1: Table S5). The SSRs identified in this study would facilitate genetic linkage map construction and marker-assisted selection (MAS) in radish breeding programs.

### Analysis of genetic variation between three genotypes

To investigate the distributions of SNPs and InDels in genic and intergenic regions, the location of genetic variation was compared with the annotation of the reference gene models. Among the 22,412 SNPs, 11,236 (50.1%) SNPs were located in intergenic regions, 5483 (24.5%) in intron, and 5693 (25.4%) in coding sequence (CDS); and the SNP frequencies were 1 SNP/35.8, 73.3 and 70.6 kb, respectively (Table 3). For SNPs in the CDS regions, the maximum number of 4895 was detected in ‘NAU-RG’ and ‘NAU-YH’ comparison, with a frequency of 1 SNP/82.1 kb; the lowest number of 4536 SNPs was observed in ‘NAU-LB’ vs ‘NAU-RG’ comparison with a frequency of 1 SNP/88.6 kb. Moreover, 1413 (15%) InDels were located in intergenic regions, 1135 (12%) in intron, 6888 (73%) in CDS, with a frequency of 1 InDel/58.4, 354.2 and 284.5 kb, respectively. The variations of InDel in the CDS regions, the maximum (6301, 1 InDel/0.06 Mb) and minimum (6015, 1 InDel/0.07 Mb) numbers of InDels also existed in ‘NAU-RG’ vs ‘NAU-YH’ comparison and ‘NAU-LB’ vs ‘NAU-RG’ comparison, respectively.

**Table 3:** Distribution and frequency of SNPs and InDels in functional regions of the radish genome

Category	SNPs			InDels		
	Number	Percent (%)	Frequency (per bp)	Number	Percent (%)	Frequency (per bp)
Intergenic	11,236	50.1	35,777.9	1413	14.9	284,501.1
Intron	5483	24.5	73,317.5	1135	12.1	354,185
CDS	5693	25.4	70,613	6888	73.0	58,362.4
Total	22,412	100	17,936.8	9436	100	42,593.8

**Fig. 6:**
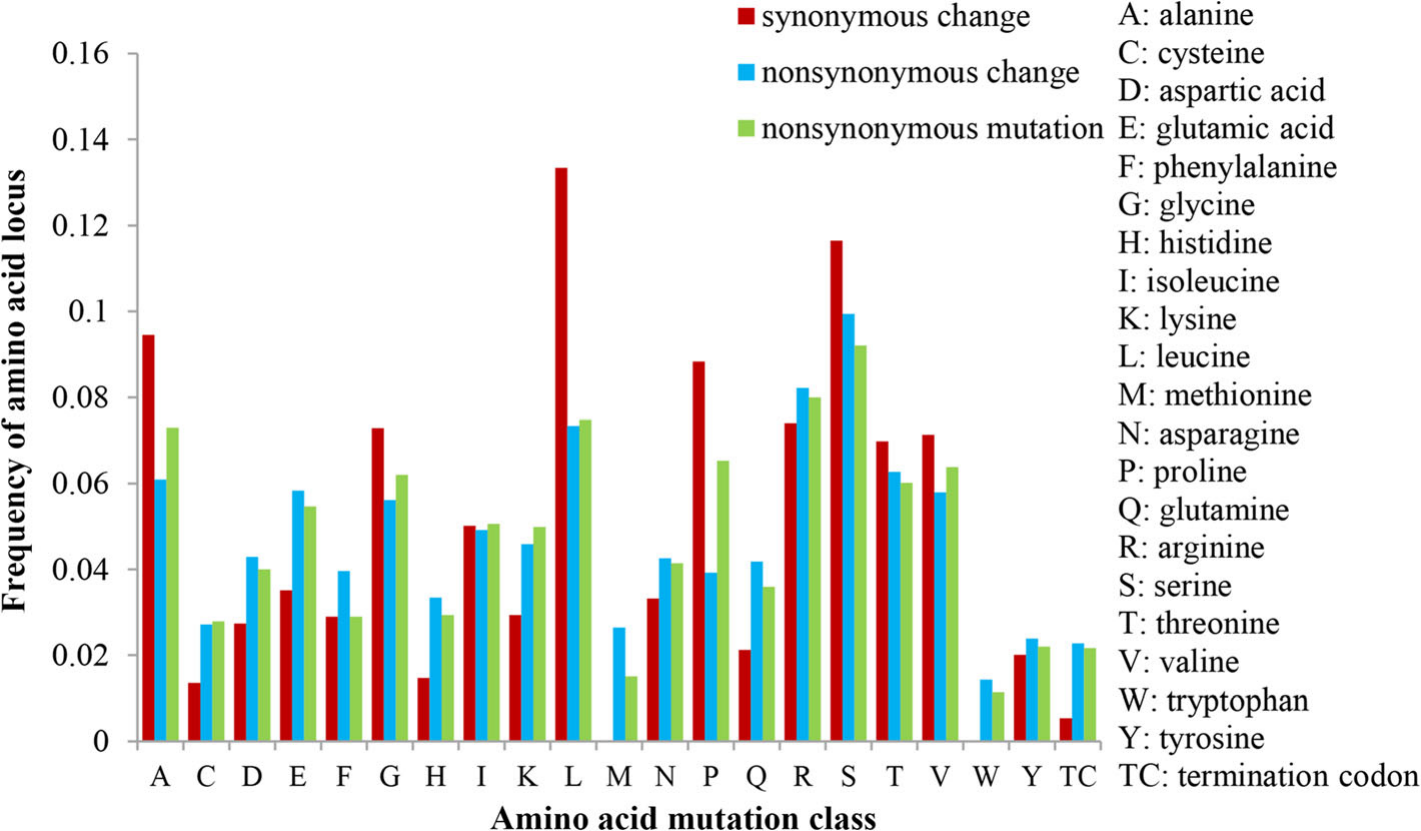
Amino acid mutations of SNP in coding regions. The vertical axis represents the frequencies of amini acid locus. The horizontal axis show the class of amnio acid identified in this study

In addition, all the detected SNPs in the coding region were categorized as synonymous or non-synonymous. A set of 5318 coding SNPs were obtained, of which 2592 (48.74%) were synonymous SNPs, whereas 2726 (51.26%) were nonsynonymous SNPs and caused amino acid changes in protein sequences. The ratio of nonsynonymous vs. synonymous SNPs was 1.05:1. The majority of synonymous SNPs were the leucine (L) (13.35%), while methionine (M) and tryptophan (W) were absent in synonymous changes. Serine (S) (9.21%) and arginine (R) (8%) were the top two types of the nonsynonymous SNPs change. For the nonsynonymous mutation, the alanine (A) (7.3%) was mutated easier than the original amino acid loci (6.09%) (Fig. 6). Moreover, the predominant proteins correlation to chromatin binding, signal transducer activity and motor activity had more variations as their encoding genes had more nonsynonymous SNPs. Among these genes containing synonymous SNPs, the predominant molecular functions of proteins were involved in structural molecular activity, DNA binding, kinase, and transporter activity (Additional file 1: Figure S2).

### Validation of markers and linkage group (LG) locations in radish

**Fig. 7:**
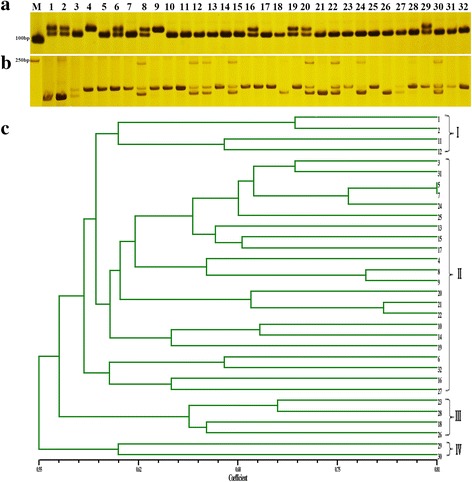
Genetic diversity analysis of 32 radish accessions with InDel markers. Amplification of 32 genotypes with InDel primers NAU InDel15 (**a**) and NAU InDel27 (**b**) by polyacrylamide gel electrophoresis, the UPGMA dendrogram of 32 radish genotypes based on 40 new InDel markers (**c**). M: 500 bp DNA ladder, the genotype name of numbers (1–32) were listed in Additional file 1: Table S1

To evaluate the accuracy and efficiency of putative polymorphisms, a total of 17 DNA sequences spanning 35 SNPs in ‘NAU-LB’, ‘NAU-YH’ and ‘NAU-RG’ were randomly selected for PCR amplification and Sanger sequencing (Additional file 8). In total, 15 primer pairs could amplify expected sizes, containing a total of 31 SNP loci, of which 26 (83.9%) SNPs revealed identifiable polymorphisms among three genotypes. A total of 180 candidate InDels with lengths greater than or equal to 3 were designed for PCR validation on polyacrylamide gels electrophoresis (PAGE) gels, of which 131 primer pairs showed distinguishable polymorphisms among three genotypes (Fig. 7a-b). However, the other 25 primer pairs showed monomorphism, 14 primer pairs exhibited unexpected size and 10 primer pairs could not successfully be amplified in three genotypes. As for the InDels with ≤2 bp could not be unambiguously discriminated on PAGE gels, a total of 20 InDels with lengths of less than 3 bp were randomly selected for Sanger sequencing (Additional file 1: Table S6), from which 9 loci were polymorphic among three genotypes. Accordingly, 140 of the 200 primer pairs (70%) revealed identifiable polymorphisms among three genotypes.

To investigate the linkage group (LG) locations of SNP and InDel markers developed in this study, the scaffolds contained SNPs and InDels were separately anchored onto the approximate location of linkage groups. For the validated SNPs and InDels, 15 SNPs and 125 InDels were aligned on radish linkage groups. A total of 140 markers were found to be unevenly distributed on 9 linkage groups (R1-R9) (Additional file 1: Figure S3). Among the 9 linkage groups, R2 contained the largest number of markers (26 markers), while only 5 markers were mapped to R3. Moreover, there were 15 and 9 markers clustered in the bottom of chromosome R2 and R4, respectively.

### Application of InDels in genetic diversity analysis of radish germplasm

To assess the value of the InDel markers, 40 InDel marker primers were further used for genetic diversity analysis among 32 worldwide radish accessions. The amplification of 40 polymorphic InDels markers generated 86 alleles across 32 germplasm accessions. The total number of alleles (Na) ranged from 2 to 4 with an average of 2.15. Expected homozygosty (HO) per locus ranged from 0 to 0.53, with an average of 0.24. Expected heterozygosity (HE) per locus ranged from 0.32 to 0.75 with an average of 0.44. The polymorphic information content (PIC) values varied from 0.09 to 0.69 and the mean PIC value was 0.40 (Table 4). The dendrogram showed that the 32 radish accessions could be divided into four major clusters with similarity coefficient varying from 0.55 to 0.81 (Fig. 7c). The cluster I comprised four Chinese accessions, most of which had late maturity characters, and the root shapes were long cylindrical. The cluster II included 22 accessions, and most of the accessions had different geographical origins, taproot colors and maturities. In this cluster, LLYB showed a high similarity with XBY, both had white phloem and xylem with medium maturity. PI358483 originated from Macedonia and PI263262 collected from Japan were divided into a subgroup, and both of them had similar taproot color and maturity. The cluster III contained four wild accessions, the RKZ was collected from China, while the PI271451, PI436536 and X50 with different taproot colors were originated from India, Guatemala and Germany, respectively. The cluster IV consisted of one Russian and Turkey accession with medium maturity.

**Table 4:** Genetic diversity analysis with InDel markers developed in radish

InDel Primer name	Scaffold	Position	Expected	Na	HO	HE	PIC	Forward primer	Reverse primer
			size (bp)						
NAU-RsInDel1	Rsa1.0_02158.1	133,381	124	2	0.27	0.36	0.38	TCCAGCACCAAGAACTAT	TAAAACACCACCAAAAGG
NAU-RsInDel2	Rsa1.0_04545.1	9016	154	2	0.27	0.51	0.46	GTCGGATTTGGTAAAGAGG	CGTCCCTGAACTGGTCATA
NAU-RsInDel3	Rsa1.0_00949.1	39,724	141	2	0.53	0.47	0.44	CATCTTCGGGATCAACGG	ATCGGACAACTCAAAACCAACT
NAU-RsInDel4	Rsa1.0_00251.1	287,457	180	2	0	0.5	0.49	AGACTGCCGAGTATCAAT	AATCGCTGGAGAAGAAAT
NAU-RsInDel5	Rsa1.0_03247.1	13,547	96	2	0.16	0.25	0.26	AGTGTCTTTGCGGCATCT	TAGGGTTTGCGATTTGGA
NAU-RsInDel6	Rsa1.0_00071.1	141,781	111	2	0.25	0.5	0.51	TGAGGAAACAGAACAAGA	GCTTAGGCTTAGCATTAT
NAU-RsInDel7	Rsa1.0_00660.1	52,677	175	2	0.16	0.43	0.38	TGCTATCAAAACGCAAAT	CAAGGAAAATAAAACTATGGAG
NAU-RsInDel8	Rsa1.0_01209.1	39,624	112	2	0.07	0.43	0.41	ATCATCAATCTCGCTCTTT	AGACTATCACCTCCTCTGC
NAU-RsInDel9	Rsa1.0_01100.1	52,433	153	2	0.31	0.35	0.28	AGCAGCCAGTGAGATTAG	ACACGGTATTTTCCAACC
NAU-RsInDel10	Rsa1.0_00232.1	38,667	162	2	0.52	0.51	0.42	TAGTGAGTGAAGACACCAAC	TTAAAGCTGCAGAAGAAG
NAU-RsInDel11	Rsa1.0_01156.1	25,754	165	2	0.1	0.34	0.32	CCCTCTGCTTTCATCCTT	TCTCCGCTGACCACAATT
NAU-RsInDel12	Rsa1.0_15591.1	936	140	2	0.39	0.39	0.35	TACTCCAAATTCGCATCA	TCATCGGAAGGTAAGACG
NAU-RsInDel13	Rsa1.0_01184.1	26,764	177	2	0.07	0.29	0.37	ATGATTCTCCATCCAAGC	GTCGCATTTTCATTTCCT
NAU-RsInDel14	Rsa1.0_03334.1	18,880	127	4	0.41	0.75	0.69	CTACTTGTCCGAGCTTCA	TAACTCCCGTTGTTGATA
NAU-RsInDel15	Rsa1.0_27069.1	235	109	2	0.25	0.31	0.26	TTATGAGAAACGTCAAGG	AGTGGAGAAAGGAACAAG
NAU-RsInDel16	Rsa1.0_20672.1	478	172	3	0.19	0.51	0.45	TAGAGGGTGAAACGCAGGAA	TTGGGAAGTGGAACAAAGCA
NAU-RsInDel17	Rsa1.0_00250.1	39,440	164	2	0.19	0.36	0.33	ATTTCAACAGATAAACCGACAC	GCCTGAGCCCATCAACTA
NAU-RsInDel18	Rsa1.0_02411.1	6272	178	2	0.06	0.48	0.41	GGGAACCCAACAACCTAT	TTATTCGACGGCACAAAC
NAU-RsInDel19	Rsa1.0_00016.1	354,003	162	2	0.46	0.46	0.53	TCCAAGGCTAGTAGAAAC	TGACATCCAACAAAGACA
NAU-RsInDel20	Rsa1.0_01401.1	93	134	2	0.34	0.47	0.35	AAATAAGACACGAAACCCTG	ATCGAAACTCCTCCCTCC
NAU-RsInDel21	Rsa1.0_01219.1	21,510	148	2	0.13	0.5	0.45	ACAACAACGGAGACTTGG	GTGAACCTGTCGGCTATG
NAU-RsInDel22	Rsa1.0_00816.1	110,085	145	2	0.17	0.48	0.44	GTCCGAACAAAAGGTGAGATT	ACTATTGATTTGATTTGGGTTGTG
NAU-RsInDel23	Rsa1.0_00744.1	53,460	145	2	0.37	0.44	0.42	TCTCAAGGACCCAACACTACA	TTGCAAAACTGGAAAAGGAA
NAU-RsInDel24	Rsa1.0_02097.1	17,223	124	2	0.19	0.42	0.37	TAAAATTGATAAACCTGCTC	CTGTGATTCTGAACCTCC
NAU-RsInDel25	Rsa1.0_01975.1	14,276	163	2	0.03	0.03	0.09	CCAGAAAACGAATGAAAAACTACT	AGAAACACATGGCTGAGAGGA
NAU-RsInDel26	Rsa1.0_00101.1	211,799	180	2	0.34	0.5	0.37	CAGCGAAACCACAAACACAA	GCTAAGTCTCCCTCCTCCATT
NAU-RsInDel27	Rsa1.0_04806.1	7466	184	2	0.31	0.41	0.32	TGTATTAACCACCACCAT	CACGAAAGATAACAACTCAA
NAU-RsInDel28	Rsa1.0_04167.1	18,003	200	2	0.19	0.35	0.28	TCTCACCCCAGCCACTTT	TATCCCACCTGTCTCGTT
NAU-RsInDel29	Rsa1.0_02690.1	16,462	165	2	0.25	0.51	0.38	AATCTTCCTGCGCCACAT	TCGTTTGCCTTCTCCTCA
NAU-RsInDel30	Rsa1.0_08988.1	3062	105	2	0.23	0.49	0.41	AGAGGGAAGCCACTATCA	TTCCGAAGATTAGATGAGAT
NAU-RsInDel31	Rsa1.0_14009.1	2697	189	3	0.26	0.46	0.42	TCTTGTTCTTTGGGTAAT	AAGGACTCAATCTATGGC
NAU-RsInDel32	Rsa1.0_02642.1	14,660	104	3	0.03	0.48	0.5	TACAATAAAACCCTCAAG	AAGTGGTGATAAGGAAAC
NAU-RsInDel33	Rsa1.0_02467.1	15,972	131	2	0.03	0.5	0.45	CAGATAAAGCCAAAAGTC	ATCTGTTTTACTTGGAGC
NAU-RsInDel34	Rsa1.0_02467.1	15,842	130	3	0.28	0.57	0.49	TCTCAAACCAAGGAAACC	ACGAAGAACTACGAGGCT
NAU-RsInDel35	Rsa1.0_00012.1	70,110	193	2	0.41	0.5	0.37	TTTTCGAGAAGAAGAAAGTC	GGACAAATACTGCCACAT
NAU-RsInDel36	Rsa1.0_00042.1	55,669	124	2	0.28	0.48	0.36	TAAAAGCCAATACAGAAG	CACAGACGAAAGACCATA
NAU-RsInDel37	Rsa1.0_00105.1	127,102	105	2	0.29	0.5	0.41	GGAGGAAGACGAGTTGAT	TGAGACGTTTTGGAAGTG
NAU-RsInDel38	Rsa1.0_01482.1	15,564	102	2	0.26	0.41	0.49	TAAAATAACAAATCACTCCCTC	CTAACCCTAATCGCACCC
NAU-RsInDel39	Rsa1.0_00816.1	109,998	140	2	0.25	0.51	0.38	CTCACAACCCAAATCAAA	TACCTGGAGGAAGGAAAG
NAU-RsInDel40	Rsa1.0_04618.1	3100	117	2	0.47	0.49	0.45	GAAGAGCTGGAGAAGGAA	AGGCCAACTGCTAGTAGATT

## Discussion

Alternative splicing emerged as a key scheme in multicellular eukaryotes to enhance the proteome diversity [38]. SNP and InDel markers played important roles in molecular breeding program, which had been widely utilized in high-density genetic map construction, genome-wide association mapping and marker-assisted selection [20, 21]. Although several molecular markers had been successfully developed in radish [28, 29], the number of SNP and InDel markers were still limited for genetic diversity analysis and genetic mapping. To better systematically characterize the overall transcriptional landscape in radish, the identification and development of large-scale AS, SNP and InDel markers is needed for future genetic studies. In this study, a total of 177,540 AS events, 22,412 SNPs, 9436 InDels and 43,680 SSRs were successfully detected from three transcriptomes sequencing data of radish. Moreover, 40 InDel markers were further applied for genetic diversity analysis among 32 different radish accessions. To the best of our knowledge, this is the first report on identification of AS events, SNPs and InDels using comparative transcriptomic analysis in radish.

### Alternative splicing plays a crucial role in transcriptional regulation

RNA sequencing had been widely used for identification of alternative splicing levels in plants [23, 39]. In this study, a genome-wide identification and characterization of alternative splicing in radish was conducted by three transcriptome datasets. It was found that 59.4% of the total radish genes were alternatively spliced, and the frequency was corroborated with previous findings in *Arabidopsis* [24] and tomato [26]. It was commonly accepted that IR is the major type of AS in plants [23, 25]. IR is the most frequent AS type in radish, while ES is the least type (2.4% of the total events). In *Arabidopsis*, the fewer number of ES was caused by multiple exons skipped together and exon skipping utilized alternative 5′ and/or 3′ splice sites [4]. The UDP-galactose 4-epimerase as a member of the short chain dehydrogenase superfamily were underwent splicing variants in *Aspergillus flavus* [40]. In this study, *Rsa1.0_16819.1_g00001.1*, encoding the UDP-D-galactose 4-epimerase 1 also showed alternative splicing. *Rsa1.0_01846.1_g00001.1*, encoding the 2-oxoglutarate (2OG) and Fe (II)-dependent oxygenase superfamily protein showed tissue-specific expression, which was coincided with previously studies that a few genes exhibited tissue-specific splicing variants in plants [23, 26]. Recent studies revealed that different transcripts produced from individual multi-exon genes could accelerate genome evolution through generating new functions [3]. Three *BrFLC1* alleles with alternative splicing patterns greatly contributed to flowering-time variations in *B. rapa* [41], indicating that the AS has important biological consequences in plants. Together, the identification of AS patterns in genes would facilitate understanding the transcriptional complexity in root vegetable crops.

### Features of SNPs and InDels by comparative transcriptomic analysis

It has increasingly been proven that SNPs and InDels can potentially be linked to functional genes involved in important agronomic traits [8, 17]. Comparative transcriptomic analysis has been widely applied for identification of SNPs and InDels in many plants, such as *B. napus* [9], tomato [11] and Petunia [42]. In this study, the SNP and InDel density was 1 SNP/17.9 kb and 1 InDel/42.6 kb, respectively, which was lower than previously described average occurrence of 3.9/kb by resequencing 93 radish F_2_ individuals [29]. Generally, genomic DNA sequences had higher SNP frequency than those observed in the transcribed regions. Additionally, the SNPs and InDels within the coding regions are more likely to contribute to agronomic phenotypes. Among pairwise comparisons, the largest numbers of SNPs and InDels were present in the ‘NAU-RG’ vs ‘NAU-YH’ comparison, which suggested that ‘NAU-RG’ vs ‘NAU-YH’ had a far relationship than the other two comparisons. Moreover, the transition-transversion ratio (1.41:1) presented in this study was similar to the previous findings in melon [43] and Petunia [42]. The Ts/Tv bias could be explained by the fact that cytosine-guanine (CpG) dinucleotides exhibited the high transition frequencies after methylation [44]. Notably, a total of 13 InDel types were found in these three genotypes, and the two most common types were single- and bi-nucleotide InDels, which have also been reported in several plant species including *B. rapa*, cotton and sesame [45–47].

In the current study, the identified SNPs/InDels heterozygotes (81.5%/80.5%) were predominate as compared with homozygotes (18.5%/19.5%), confirming that the high heterozygosity rate was found in three accessions. Meanwhile, the clean reads uniquely mapped to the radish reference genome were less than 80% in each transcriptome, which was similar to previously reported results that heterozygous polymorphisms had been supported by a low proportion of uniquely mapped reads [48]. Previous studies have identified a number of heterozygous single-nucleotide variants (SNVs) in *Puccinia striiformis* f. sp*. Tritici* (*Pst*)-CY32 genome, and some of the predicted genes were located in the heterozygous regions [49]. Wang et al. [50] revealed that heterozygous alleles played significant roles in restoring male fertility of cytoplasmic male-sterile in radish. Therefore, the results would provide useful information for the research of genome heterozygosity and its molecular function in radish.

Based on the genome position of SNPs and InDels, the sequence region types were classified. The SNPs frequency was enriched almost equally between genic (49.9%) and intergenic (50.1%) regions. Approximately 85% of the total InDels was identified in genic regions, which was similar to previously reported in rice and sesame [47, 51]. It has been extensively shown that SNPs/InDels in the coding region were most influential variations, and mainly lead to non-function of protein [48, 52]. Notably, 73% of all InDels were identified in the CDS regions, which could cause frameshift mutation that results in the changes of gene function. Numerous studies demonstrated that non-synonymous played significant roles in amino acid variation in the protein product of genes [53]. The total numbers of nonsynonymous SNPs and synonymous SNPs were 2592 and 2726, the nonsynonymous to synonymous ratio of 1.05 exhibited a medium value compared with *Arabidopsis* (0.83) [54] and rice (1.29) [55]. Together, these results illustrated the identified SNPs and InDels would be valuable molecular tool for identification of causal gene mutations in radish.

### Characterization and polymorphism of SNP and InDel markers

In this study, a total of 35 SNPs and 200 InDels identified from three transcriptome sequences of the ‘NAU-RG’, ‘NAU-LB’ and ‘NAU-YH’ were employed for designing PCR primer. The polymorphism rate of SNP/InDel (83.9%/70%) among these three radish genotypes was in accordance with previously reported results [42, 45]. Obviously, the polymorphism ratios for transcriptome derived SNPs was 84.3% by selecting a 51 SNPs set in *Petunia* spp. [42]. A large-scale ESTs sequence data from different cotton species were used to identify putative InDels, of which 71.1% InDels were showed loci polymorphisms [46]. Several identified markers amplified with unexpected bands and failed amplification, which might be related to the presence of large introns in genomic sequence [56]. Most of the InDel (87.5%, 35/40) markers produced two allele products, which is similar to the previous results in tomato [8]. Moreover, the average PIC value of these 40 InDels markers was 0.40, which is relatively higher than the PIC value calculated in sesame [47] but lower than that in trifoliate orange [48], indicating that the moderate level of polymorphism of the developed markers would greatly facilitate genetic diversity analysis in radish.

### Availability of InDels in radish genetic diversity analysis

InDels are important tools for a wide range of genetics and genomic studies. In this study, phylogenetic analysis revealed that 32 radish accessions were grouped into four distinct clusters, thereby demonstrating the availability of the InDel markers to differentiate the cultivars. Besides, the distribution of radish cultivars was not strictly base on the geographical origins, which is consistent with previous studies in genus *Oryza* [57]. It is generally accepted that InDel markers display higher interspecies differentiation than SSR markers [58]. Currently, the efficient differentiation of the cultivars and wild radish by InDel markers in third and fourth main cluster was higher than previous reported SSR marker in 32 radish accessions [59]. However, most of Chinese cultivars were clustered in the same subgroup in the dendrogram, indicating that the genetic relationship of Chinese radish cultivars is still narrow. Therefore, it is important to introduce more exotic genotype for expanding the genetic basis of radish.

## Conclusions

In summary, this is the first report on transcriptome-based characterization of AS events, SNP and InDel markers in radish. Totally, 56,530 AS events were accounted for 59.4% of the total radish genes among these three transcriptomes. Moreover, 22,412 SNPs, 9436 InDels and 43,680 SSRs were found in all the three genotypes. Among the total SNPs located in coding regions, the percentage of nonsynonymous and synonymous SNPs were 48.74% and 51.26%, respectively. The polymorphism rate of SNPs and InDels was verified among three radish accessions, and 83.9% of SNPs and 70% of InDels exhibited polymorphism. Totally, 15 SNPs and 125 InDels were found to be unevenly distributed on radish nine linkage groups. Germplasms were successfully differentiated by several newly developed InDel markers. Taken together, our results could provide a better understanding of the radish transcriptome complexity, and these newly developed molecular markers would be excellent tool for construction of linkage map, identification of candidate genes for interest traits, and marker-assisted selection in radish breeding programs.

## Methods

### Plant materials and DNA extraction

Seeds of radish advanced inbred line, ‘NAU-RG’, ‘NAU-LB’, ‘NAU-YH’ and other 32 radish accessions with different root colors and origins (Additional file 1: Table S1) were soaked, surface-sterilized and incubated for 3 days. The germinated seeds were grown in plastic pots and cultured in greenhouse under 25 °C/14 h light and 18 °C/10 h dark. Young leaves of 30-day-old seedlings were collected for DNA extraction. Genomic DNA of all accessions were extracted from young leaves using a modified CTAB procedure [60]. Then DNA samples were diluted to a final concentration of 10 ng μl^−1^ with 1 × TE buffer for further use.

### Transcriptome sequences and mapping reads to reference genome

Three transcriptome data, sequencing from roots of genotypes ‘NAU-YH’ and ‘NAU-RG’ (NCBI accession ID No. SRX707630 and SRX316199) [31, 32], leaves of ‘NAU-LB’ (NCBI accession ID No. SRX1671013) [33], were collected from our previously reports. The radish reference genome sequence and gene model annotation files were downloaded from the available genome website (ftp://ftp.kazusa.or.jp/pub/radish/). Index of the reference genome was built using Bowtie v2.2.3, and paired-end clean reads mapped to the radish reference genome using TopHat v2.0.12 [34]. TopHat can generate a database of splice junctions based on the gene model annotation file.

### Identification and validation of AS events

Splice junctions were identified using TopHat program, and then filtered the junctions with reads number less than three. The known and novel transcripts were identified by Cufflinks v2.1.1 Reference Annotation Based Transcript (RABT) assembly method [61]. The final transcript annotations in a GTF format file were used to identify AS events using ASTALAVISTA program with default settings [35]. Five types including IR, A3SS, A5SS, ES and others that contain more than one of the four basic types were analyzed as previously described [4]. The mapping results of AS events were visualized using the Integrative Genomics Viewer (IGV) [62]. Alternative splicing isoforms were quantified between the three genotypes using the MATS program [36]. The differentially spliced events were defined with at least 10% change in exon inclusion level and false discovery rate (FDR) of less than 0.05. The splicing events were represented with the sashimi plots [63].

Total RNA was isolated from leaves and roots of ‘NAU-RG’, ‘NAU-LB’, and ‘NAU-YH’ with Trizol reagent (Invitrogen, Carlsbad, CA, USA) according to the manufacturer’s instructions. The RNAs were treated with RNase-free DNase I (Takara, Japan), and reverse-transcribed into cDNAs using SuperScript II reverse transcriptase (Invitrogen). The primer information is listed in Additional file 1: Table S3. *RsActin* was used as the internal control, and PCR products were visualized in agarose gel stained by ethidium bromide (EB).

### Identification of SNPs, InDels and SSRs

The Picard-tools v1.96 and samtools v0.1.18 programs were used to sort and mark duplicated reads as well as reorder the alignment results of each sample [64]. SNP and InDel calling were carried out using the Genome Analysis Toolkit (GATK, version v3.2) [37], and the variant were filtered as follows: low quality scores (QUAL) is less than 30 and quality by depth (QD) is less than 5. Only the putative SNPs or InDels with 10 or more reads coverage were recorded to reduce the numbers of false positive SNPs. The localization of SNPs and InDels was performed based on the annotation of gene models of the radish genome. Synonymous and non-synonymous substitutions of the SNPs were determined using the SNPEff program [65]. Blast2GO program was applied to achieve Gene Ontology analysis on the genes contained SNPs and InDels.

To generate the reference transcriptome sequences, a de novo assembly of three transcriptome data was performed using Trinity program [66]. The potential SSRs were identified from the assembled unigenes using the MISA (microsatellite identification tool) program [67]. The number of repetitive units with a minimum of 10, 6, 5, 5, 5 and 5 were defined for the mono- to hexa-nucleotide, respectively.

### Primer design and validation of DNA polymorphism

Primer pairs flanking the SNPs or InDels were designed using Primer Premier 5.0 program. The length of primer pairs ranged from 18 to 24 bp, while the predicted product sizes were varied from 80 bp to 400 bp. The *Tm* value was restricted to between 50 and 60 °C. PCR was carried out in a 15 μl reaction volume containing 10 ng of template DNA, 2.0 mM of MgCl_2_, 0.2 mM dNTPs, 0.1 μM of each primer and 0.5 U Taq DNA polymerase (TaKaRa Bio Inc., Dalian, China). The PCR procedure comprised an initial denaturation at 94 °C for 3 min, followed by 35 cycles of 94 °C for 50 s, 56 °C for 50 s and 72 °C for 1 min, with a final extension at 72 °C for 10 min. The SNP amplified PCR products were sequenced by Sanger method and analyzed by BioEdit v 7.0.5.3 (http://www.mbio.ncsu.edu/BioEdit/ bioedit.html). The InDel amplified products were separated on 8% PAGE and visualized with silver staining as described previously [68]. The validated SNPs and InDels were mapped on the prior dense genetic maps of radish according to their approximate positions on the linkage group locations using MapInspect software (http://mapinspect.software.informer.com/) [30].

### Data analysis

To estimate the allelic variation of InDel markers in 32 radish accessions, POPgene program (v1.32) was employed to estimate the total number of alleles, expected homozygosty and expected heterozygosity [48]. The polymorphic information content value for the InDel marker was calculated using Power Marker v3.0 [69]. The 0 – 1 data matrix was further used to calculate the coefficients of genetic similarity among all the accessions using the SIMQUAL program of NTSYS-pc software. The dendrogram was constructed basing on the unweighted pair-group method with arithmetic averages (UPGMA) in the SAHN module of NTSYS-pc software [59].

## Additional files

Additional file 1: Table S1. Radish genotypes used for genetic diversity analysis in this study. **Table S2.** Numbers of identified splice junction reads in three transcriptomes. **Table S3.** RT-PCR experimental validation of AS events. **Table S4.** Distribution and frequency of InDels in radish. **Table S5.** Distribution of mono- to tetranucleotide repeats in radish ranscriptome. **Table S6.** The InDels validated by Sanger sequencing among three genotypes. **Figure S1.** Distribution of AS events in radish genome. **Figure S2.** Synonymous and non-synonymous SNP distribution in 16 major protein molecular functions. **Figure S3.** Linkage group (LG) localization of SNP and InDel markers in the radish genome. The bar on the left shows the marker positions [cM], marker names are shown on the left of each linkage group. (DOCX 1013 kb)

Additional file 2: The information of detected alternative splicing (AS) events in radish. (XLSX 5752 kb)

Additional file 3: The differential alternative splicing events between three genotypes (XLSX 740 kb)

Additional file 4: List of novel transcripts detected in the genotypes ‘NAU-YH’, ‘NAU-LB’ and ‘NAU-RG’. (XLSX 523 kb)

Additional file 5: Putative single nucleotide polymorphisms (SNPs) in the genotypes ‘NAU-YH’, ‘NAU-LB’ and ‘NAU-RG’. (XLSX 1184 kb)

Additional file 6: Putative insertion/deletion (InDel) in the genotypes ‘NAU-YH’, ‘NAU-LB’ and ‘NAU-RG’. (XLSX 517 kb)

Additional file 7: The detailed information of putative SSR markers identified in radish. (XLSX 2112 kb)

Additional file 8: Primer sequences used for SNPs validation between ‘NAU-YH’, ‘NAU-LB’ and ‘NAU-RG’ by Sanger sequencing. (XLSX 54 kb)

## Abbreviations

A3SS: Alternative 3′ splice site donor
A5SS: Alternative 5′ splice site donor
AS: Alternative splicing
CDS: Coding sequence
ES: Exon skipping
HE: Expected heterozygosity
HO: Expected homozygosity
InDel: Insertion/deletion
IR: Intron retention
LG: Linkage group
Na: Number of alleles
NCBI: National center for biotechnology information
PAGE: Polyacrylamide gel electrophoresis
PIC: Polymorphic information content
SNP: Single nucleotide polymorphism
SSR: Simple sequence repeat
Ts: Transition
Tv: Transversion
UPGMA: Unweighted pair-group method with arithmetic averages
