# Supplementary material for: Comparative transcriptomics uncovers alternative splicing and molecular marker development in radish (Raphanus sativus L.)
Source: BMC Genomics. 2017 Jul 3;18:505. doi: 10.1186/s12864-017-3874-4 (PMC5496183; doi:10.1186/s12864-017-3874-4)
Supplement: Supplementary file 1 — Radish genotypes used for genetic diversity analysis in this study. Table S2. Numbers of identified splice junction reads in three transcriptomes. Table S3. RT-PCR experimental validation of AS events. Table S4. Distribution and frequency of InDels in radish. Table S5. Distribution of mono- to tetranucleotide repeats in radish ranscriptome. Table S6. The InDels validated by Sanger sequencing among three genotypes. Figure S1. Distribution of AS events in radish genome. Figure S2. Synonymous and non-synonymous SNP distribution in 16 major protein molecular functions. Figure S3. Linkage group (LG) localization of SNP and InDel markers in the radish genome. The bar on the left shows the marker positions [cM], marker names are shown on the left of each linkage group. (DOCX 1013 kb) [file 12864_2017_3874_MOESM1_ESM.docx]

**Table S1.** Radish genotypes used for genetic diversity analysis in this study.

| No. | Genotype | Origin | Skin color | Root shape | Maturity |
| --- | --- | --- | --- | --- | --- |
| 1 | BYC | Korea, South | White | Long | Medium-late |
| 2 | CQDG | Osaka, Japan | White | Long | Late |
| 3 | LLDY13 | Nanchang, China | White | Long | Medium-late |
| 4 | CBL | Korea, South | White | Long | Late |
| 5 | LLYB | Nanjing, China | White | Long | Medium |
| 6 | LLZDC | Nanjing, China | White | Long | Late |
| 7 | XBY | Shanxi, China | White | Long | Medium-late |
| 8 | PI358483 | Macedonia | White | Top | Late |
| 9 | PI263262 | Osaka, Japan | White | Long | Late |
| 10 | YZH | Mianyang, China | Red | Long | Medium |
| 11 | QTDHP | Wuhan, China | Red | Globe-long | Late |
| 12 | QTCXH | Nanjing, China | Red | Globe-long | Medium |
| 13 | QSH | Mianyang, China | White-red | Globe-long-half long | Early-mediu |
| 14 | JYCH | Taizhou, China | Red | Globe | Early |
| 15 | XHW | Hong Kang, China | Red | Long | Early |
| 16 | JH1 | Sichuan, China | Red | Globe | Medium |
| 17 | NJH | Nanjing, China | Red | Globe | Medium |
| 18 | PI436536 | Guatemala | Red | Top | Early |
| 19 | LLQ | Nanjing, China | Green | Top | Early |
| 20 | LTQ | Ningxia, China | Green | Half long | Medium |
| 21 | LWQ | Xuzhou, China | Green | Globe | Late |
| 22 | WXQ | Weifang, China | Green | Half | Medium |
| 23 | RKZ | Xizhang, China | Black | Conical | Late |
| 24 | MEC | Yongan, China | White | Long | Early |
| 25 | 501 | Dalian, China | Green | Globe-top | Early |
| 26 | X50 | Germany | Purple | Long | Early |
| 27 | PI381011 | Washington,USA | White | Long | Early |
| 28 | PI271451 | Gujarat, India | White | Long | Early |
| 29 | PI262942 | Leningrad, Russian | Red-pink-white-purple | Globe-top-half long | Medium |
| 30 | PI121018 | Turkey | Purple | Top-long | Medium |
| 31 | PI183242 | Egypt | White | Long | Early |
| 32 | PI140428 | Iran | White | Top | Early |

**Table S2.** Numbers of identified splice junction reads in three transcriptomes.

|  | Total (unique) | NAU-RG | NAU-LB | NAU-YH |
| --- | --- | --- | --- | --- |
| identified junctions (reads-depth >=3) | 170,086 | 131,711 | 135,732 | 133,593 |
| containing junctions have been annotated | 131,106 | 106,741 | 113,026 | 109,593 |
| novel junctions | 38,980 | 25,030 | 22,672 | 24,000 |

**Table S3.** RT-PCR experimental validation of AS events.

| Event_id | Forward primer | Reverse primer | Ref_id |
| --- | --- | --- | --- |
| 1003168 | TTCCATCTTTCCCACCAT | AGCACCGACTGCTACTGC | gene:Rsa1.0_00033.1_g00004.1,mRNA:Rsa1.0_00033.1_g00004.1 |
| 1074935 | AAAGGTGGTACGGAGGAA | TAGCAGAAGCAGCAGTCA | CUFF.46442.3,mRNA:Rsa1.0_08877.1_g00001.1 |
| 1089654 | TGCTGGAATAGCTTCACA | TGCTGGAATAGCTTCACA | gene:Rsa1.0_55628.1_g00001.1,mRNA:Rsa1.0_55628.1_g00001.1 |
| 1045189 | CAAACCGATAATCAGAGC | CAATGGGCTAATAAGGAT | CUFF.26479.4/CUFF.26479.5/mRNA:Rsa1.0_01846.1_g00001.1,CUFF.26479.3 |
| 1042232 | GTTGTTCTTGAGGCTTAC | TGTGGTGGTTATTCATCT | CUFF.24679.3/CUFF.24679.4/CUFF.24679.5,mRNA:Rsa1.0_01595.1_g00005.1 |
| 1075044 | CCTGGAAGGACAGAACCC | GACGCAGAAGATGAAGAAGAGT | mRNA:Rsa1.0_09443.1_g00002.1,CUFF.46685.3 |
| 1073106 | GAGGCGATGCTGTTAGTA | TCAAAGCGATCTTCTTGC | mRNA:Rsa1.0_08198.1_g00002.1,CUFF.45085.1 |
| 1081797 | TTTCGCAATGAATCCAATC | TCAACTGAGCCAAGTGTT | mRNA:Rsa1.0_16819.1_g00001.1,CUFF.52448.3 |
| 1079451 | ATGCCGAGGAGTTGAAGA | TTGGGAGAAGTGGTGTTATT | mRNA:Rsa1.0_13815.1_g00001.1,CUFF.50458.1/CUFF.50458.5 |
| LBvsRG | GGAGAAGTATGGAGGGTT | GTTAGCAACAAAGGAGCA | Rsa1.0_01282.1_g00001.1 |

**Table S4.** Distribution and frequency of InDels in radish.

| InDel size (bp) | Number | Frequence (%) |
| --- | --- | --- |
| 1 | 7807 | 82.74% |
| 2 | 1390 | 14.73% |
| 3 | 122 | 1.29% |
| 4 | 18 | 0.19% |
| 5 | 21 | 0.22% |
| 6 | 31 | 0.33% |
| 7 | 15 | 0.16% |
| 8 | 12 | 0.13% |
| 9 | 10 | 0.11% |
| 10 | 6 | 0.06% |
| 11 | 1 | 0.01% |
| 12 | 2 | 0.02% |
| 13 | 1 | 0.01% |

**Table S5.** Distribution of mono- to tetranucleotide repeats in radish ranscriptome

| **Repeat type** | **Motif** | **5** | **6** | **7** | **8** | **9** | **10** | **11** | **12** | **>=13** | **Total** |
| --- | --- | --- | --- | --- | --- | --- | --- | --- | --- | --- | --- |
| **Mononucleotide** | A/T | 0 | 0 | 0 | 0 | 0 | 8776 | 3883 | 2169 | 6414 | 21242 |
|  | C/G | 0 | 0 | 0 | 0 | 0 | 106 | 56 | 39 | 116 | 317 |
| **Dinucleotide** | AG/CT | 0 | 1280 | 857 | 579 | 395 | 326 | 201 | 190 | 475 | 4303 |
|  | GA/TC | 0 | 1490 | 950 | 526 | 371 | 243 | 209 | 150 | 355 | 4294 |
|  | AT/TA | 0 | 414 | 210 | 136 | 103 | 61 | 36 | 19 | 57 | 1036 |
|  | AC/GT | 0 | 153 | 114 | 80 | 35 | 36 | 17 | 18 | 12 | 465 |
|  | CA/TG | 0 | 152 | 145 | 47 | 27 | 20 | 7 | 13 | 18 | 429 |
|  | CG/CG | 0 | 2 | 0 | 0 | 0 | 0 | 0 | 0 | 0 | 2 |
| **Tetranucleotide** | AGA/TCT | 729 | 296 | 159 | 80 | 7 | 27 | 7 | 6 | 23 | 1334 |
|  | GAA/TCC | 694 | 316 | 115 | 91 | 16 | 26 | 8 | 8 | 17 | 1291 |
|  | AAG/CTT | 499 | 235 | 127 | 49 | 10 | 22 | 8 | 2 | 11 | 963 |
|  | CTC/GAG | 355 | 158 | 107 | 50 | 2 | 2 | 0 | 0 | 1 | 675 |
|  | ATC/GAT | 350 | 152 | 67 | 54 | 10 | 9 | 3 | 1 | 8 | 654 |
|  | GGA/TCC | 356 | 164 | 72 | 41 | 9 | 5 | 2 | 1 | 0 | 650 |
|  | TCA/TGA | 290 | 164 | 46 | 45 | 6 | 2 | 4 | 5 | 4 | 566 |
|  | AGG/CTT | 284 | 118 | 66 | 15 | 1 | 7 | 4 | 0 | 1 | 496 |
|  | ACC/GGT | 222 | 151 | 50 | 23 | 4 | 1 | 1 | 1 | 0 | 453 |
|  | ATG/CAT | 186 | 135 | 37 | 29 | 10 | 7 | 3 | 3 | 6 | 416 |
|  | CCA/TGG | 207 | 128 | 41 | 19 | 0 | 1 | 0 | 0 | 0 | 396 |
|  | CAA/TGG | 222 | 96 | 45 | 20 | 3 | 4 | 1 | 1 | 3 | 395 |
|  | AAC/GTT | 254 | 91 | 30 | 12 | 1 | 1 | 3 | 0 | 2 | 394 |
|  | ACA/TGT | 194 | 85 | 43 | 14 | 3 | 5 | 2 | 0 | 1 | 347 |
|  | AGC/GCT | 191 | 76 | 28 | 20 | 0 | 1 | 1 | 0 | 0 | 317 |
|  | CAC/GTG | 159 | 82 | 24 | 8 | 1 | 1 | 0 | 0 | 0 | 275 |
|  | CAG/CTG | 148 | 74 | 28 | 21 | 1 | 0 | 0 | 0 | 0 | 272 |
|  | TAT/ATA | 63 | 19 | 8 | 10 | 2 | 0 | 0 | 3 | 105 | 210 |
|  | GCA/TGC | 133 | 49 | 15 | 7 | 0 | 2 | 0 | 0 | 0 | 206 |
|  | TAA/AAT | 53 | 29 | 7 | 7 | 0 | 0 | 0 | 1 | 97 | 194 |
|  | GCG/CGC | 77 | 9 | 4 | 1 | 0 | 0 | 0 | 0 | 91 | 182 |
|  | GTC/GAC | 57 | 14 | 3 | 3 | 0 | 0 | 0 | 0 | 77 | 154 |
|  | CCG/CGG | 109 | 29 | 14 | 1 | 0 | 0 | 0 | 0 | 0 | 153 |
|  | AAT/ATT | 83 | 42 | 12 | 7 | 0 | 0 | 0 | 0 | 2 | 146 |
|  | CGA/TCG | 43 | 18 | 9 | 2 | 0 | 0 | 0 | 0 | 72 | 144 |
|  | TAG/CTA | 37 | 13 | 9 | 1 | 0 | 0 | 0 | 0 | 60 | 120 |
|  | GTA/TAC | 15 | 27 | 9 | 6 | 0 | 1 | 1 | 0 | 59 | 118 |
|  | GCC/GGC | 41 | 13 | 0 | 0 | 0 | 0 | 0 | 0 | 54 | 108 |
|  | ACT/AGT | 59 | 20 | 2 | 5 | 1 | 2 | 2 | 0 | 0 | 91 |
|  | ACG/CGT | 26 | 17 | 15 | 1 | 0 | 0 | 0 | 0 | 0 | 59 |

**Table S6.** The InDels validated by Sanger sequencing among three genotypes.

| Primer | Loci | Chromosome | Position | Forward primer | Reverse primer |
| --- | --- | --- | --- | --- | --- |
| RsID1 | NAU-RsInDel181 | Rsa1.0_00141.1 | 20967 | CACAGGAACTGATAACTTGGAC | TATGGTGAGGGGTTTTGC |
|  | NAU-RsInDel182 | Rsa1.0_00141.1 | 20937 |  |  |
| RsID2 | NAU-RsInDel183 | Rsa1.0_00903.1 | 58555 | CGATGTTGATGAAGCAG | GCAAGTTTTAATGTCCGT |
|  | NAU-RsInDel184 | Rsa1.0_00903.1 | 58543 |  |  |
| RsID3 | NAU-RsInDel185 | Rsa1.0_01475.1 | 25233 | GCTGTCCGCAAAGTGTATG | ATCGGTTTCTCCAGGGTT |
|  | NAU-RsInDel186 | Rsa1.0_01475.1 | 25115 |  |  |
| RsID4 | NAU-RsInDel187 | Rsa1.0_00189.1 | 545 | ACAACGAGTAAGGGAATG | GTTTAACTACCGAGACGA |
|  | NAU-RsInDel188 | Rsa1.0_00189.1 | 670 |  |  |
| RsID5 | NAU-RsInDel189 | Rsa1.0_00190.1 | 52701 | TCATTCTTTCACCGTTAC | ATCTTTCTCCTGCTCTGT |
|  | NAU-RsInDel190 | Rsa1.0_00190.1 | 52680 |  |  |
| RsID6 | NAU-RsInDel191 | Rsa1.0_01251.1 | 35040 | CGTTGGCTCTGTGGGTCT | TCGGCAATGATTTGTGGTAA |
| RsID7 | NAU-RsInDel192 | Rsa1.0_00541.1 | 61576 | TGAAATGAGGGAAATGAATC | AGCAGGAGGAAAGACAAG |
| RsID8 | NAU-RsInDel193 | Rsa1.0_00033.1 | 705 | TTCTTACGAATATGGTGG | GTTCCTTTGTGGTTGACT |
| RsID9 | NAU-RsInDel194 | Rsa1.0_08831.1 | 4542 | ATTTTGCGTCCGAGTTTT | TTTTGGGATCATGTCTTT |
| RsID10 | NAU-RsInDel195 | Rsa1.0_09755.1 | 1599 | AAGAAGACCGAGAACTGT | TTACAAGGTAGGAGCATC |
| RsID11 | NAU-RsInDel196 | Rsa1.0_03631.1 | 3450 | CATCCTCATCACTTTTGGAG' | TGGCAGGGCTTATTGTTA |
| RsID12 | NAU-RsInDel197 | Rsa1.0_05546.1 | 5386 | CACGAGCAGTTCGTCATA | CGAGTCTCCTGTCCCATT |
| RsID13 | NAU-RsInDel198 | Rsa1.0_05871.1 | 6760 | GCCAAAGCCTAAGACTGC | TGTAATTCCACGACCAAA |
| RsID14 | NAU-RsInDel199 | Rsa1.0_03306.1 | 12126 | TGCTGATTACAGGGTGAG | CGGAGATAATAAGCCAAC |
| RsID15 | NAU-RsInDel200 | Rsa1.0_00905.1 | 24065 | TTCCTCATCGCTTTGCTC | AACTCGCCAATGTGAACG |

Position: position of the InDels on the scaffolds in radish reference genome.

**Figure S1. Distribution of AS events in radish genome.**


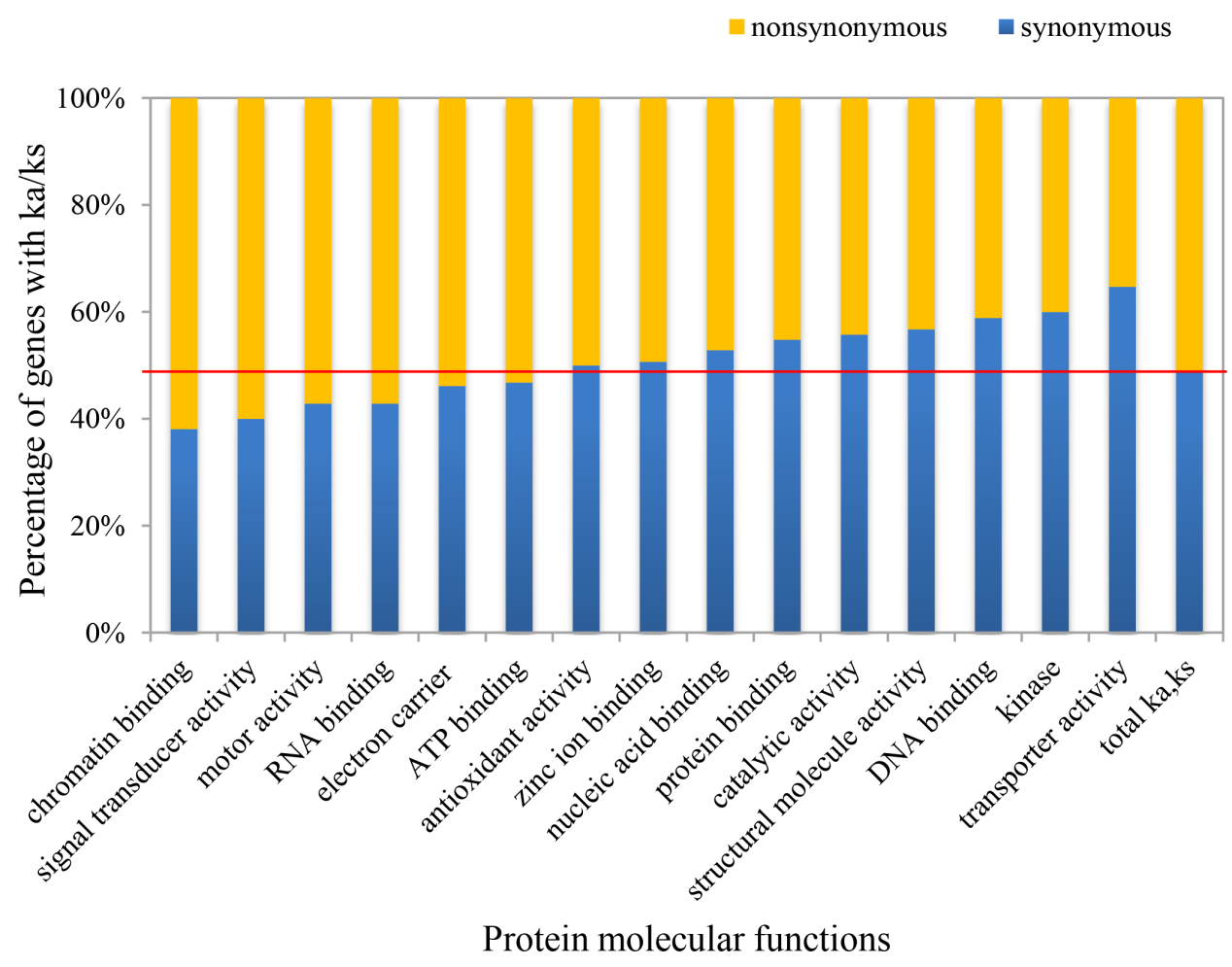


**Figure S2** Synonymous and non-synonymous SNP distribution in 16 major protein molecular functions.


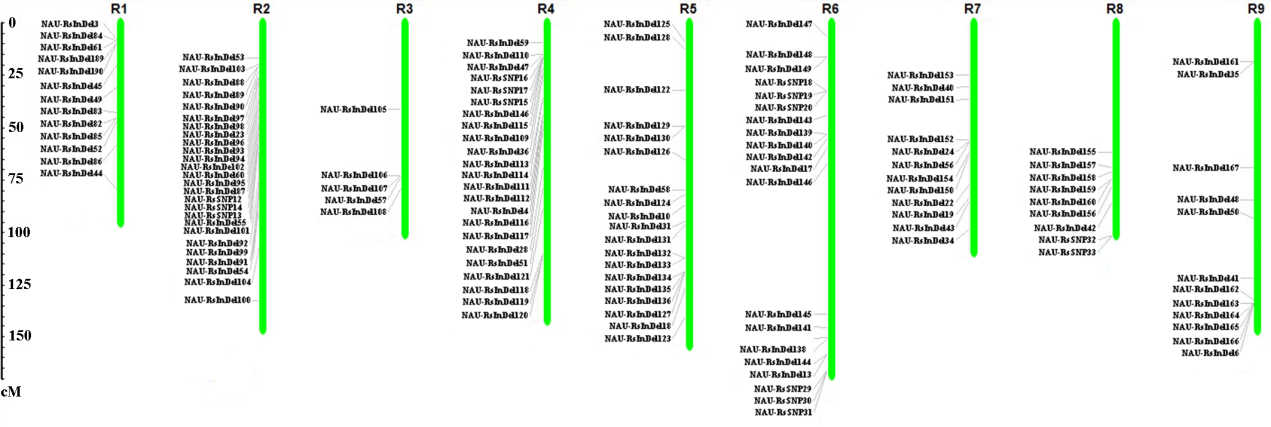


Figure S3 Linkage group (LG) localization of SNP and InDel markers in the radish genome. The bar on the left shows the marker positions [cM], marker names are shown on the left of each linkage group.
